# Safeguarding COVID-19 and cancer management: drug design and therapeutic approach

**DOI:** 10.12688/openreseurope.13841.1

**Published:** 2021-07-05

**Authors:** Nanasaheb Thorat, Sabrina Pricl, Abdul K. Parchur, Sandeep B. Somvanshi, Qifei Li, Sachin Umrao, Helen Townley

**Affiliations:** 1 Nuffield Department of Women’s & Reproductive Health, John Radcliffe Hospital, University of Oxford, Oxford, OX3 9DU, UK; 2 MolBNL@UniTS-DEA, University of Trieste, Piazzale Europa 1, Trieste, 34127, Italy; 3 Department of General Biophysics, Faculty of Biology and Environmental Protection, University of Lodz, Lodz, 90-136, Poland; 4 Radiation Oncology, Froedtert Hospital & Medical College of Wisconsin, Medical College of Wisconsin, Wisconsin, USA; 5 School of Materials Engineering, Purdue University, West Stadium Avenue, West Lafayette, USA; 6 Division of Newborn Medicine, Boston Children’s Hospital, Harvard Medical School, Boston, MA, 02115, USA; 7 Department of Therapeutic Radiology, Yale School of Medicine, Yale University, New Haven, USA; 8 Department of Engineering Science, University of Oxford, Oxford, UK

**Keywords:** Cancer, Covid 19, anticancer drugs, antiviral therapies

## Abstract

Recent clinical cohort studies have highlighted that there is a three-fold greater SARS-Cov-2 infection risk in cancer patients, and overall mortality in individuals with tumours is increased by 41% with respect to general COVID-19 patients. Thus, access to therapeutics and intensive care is compromised for people with both diseases (comorbidity) and there is risk of delayed access to diagnosis. This comorbidity has resulted in extensive burden on the treatment of patients and health care system across the globe; moreover, mortality of hospitalized patients with comorbidity is reported to be 30% higher than for individuals affected by either disease. In this data-driven review, we aim specifically to address drug discoveries and clinical data of cancer management during the COVID-19 pandemic. The review will extensively address the treatment of COVID-19/cancer comorbidity; treatment protocols and new drug discoveries, including the description of drugs currently available in clinical settings; demographic features; and COVID-19 outcomes in cancer patients worldwide.

## Plain language summary

To create an evidence-based therapeutic approach and to build clinical protocols for treating patients affected both by cancer and SARS-CoV-2 infection, systematic clinical research of the implications of acute COVID-19 and malignant neoplasms is required. In addition, a comprehensive understanding of the comorbid patient care required beyond the acute phase of COVID-19 is also urgently needed. New therapy protocols combining anticancer, antiviral, antifungal, and other supporting medications will aid in providing integrated multispecialty patient care. The most recent clinical cohort studies discussed in this review suggest that managing cancer patients infected with SARS-CoV-2 require a substantial revision of the current pharmacological treatments. Furthermore, the interpretation of cancer-relevant mechanisms engaged by the SARS-CoV-2 infection is essential for evidence-based cancer therapeutics, while the links between SARS-CoV-2 infection, tumorigenesis, and cancer patient immunosuppression must be predicted in epidemiological settings. Contextually, the adoption of highly advanced approaches such as those based on the application of artificial intelligence (AI) to COVID-19/cancer combinations could be highly beneficial, as AI can reduce the development timelines of new protocols, a critical issue in the current health emergency. Many national and international health authorities including the European Centre for Disease Prevention and Control (ECDC), the European Medicines Agency (EMA), the United States Center for Disease Control and Prevention (CDC), the National Health Service (NHS) in the United Kingdom, and the National Institute of Infectious Diseases (NIID) in Japan have issued protocol guidelines for the treatment of patients affected by cancer/COVID-19 comorbidity. All authorities specifically advise that chemotherapeutic, antiviral, and steroid doses be used in a controlled manner. Furthermore, continued usage of investigational drugs must be context-dependent and adhere to the highest clinical trial standards.

## Introduction

Severe acute respiratory syndrome coronavirus 2 (SARS-CoV-2) infection continues to spread globally at an alarming rate. The coronavirus disease 2019 (COVID-19) pandemic has dramatically affected cancer management, and has had an impact on diagnosis, treatment, and related therapeutic protocols ^1, 2^. COVID-19 has caused overall morbidity and mortality in cancer patients at an unprecedented scale. Clinical database evidence is evolving on the subacute and long-term effects of COVID-19 on cancer patients. Among all types of COVID-19 comorbidities, cancer is one of the diseases with a poorer patient outcome if compared to individuals afflicted by diabetic, cardiac, or chronic illnesses, or those without other underlying malignancies. Recent clinical studies have reported mortality rates that are significantly different among continents, ranging from 3.7% to 61.5% ^3^ for developed and underdeveloped countries, respectively. Evidence from prospective trials has suggested a link between cancer and impact of COVID-19 in comorbid patients. A cohort study including all types of cancers, with 82% of patients having solid tumors, recorded 35.5% mortality in the United Kingdom (UK) for the year 2020 ^3^. Contextually, a very recent update for the year 2021 from the COVID-19 and Cancer Consortium (CCC19) highlighted a comorbidity-related death rate of 15.8% ^4– 6^. Overall, the mortality rate of cancer patients infected with SARS-CoV-2 has been reported to vary between 13% to 40.5% in large national/international cohort studies ^7^. Factors associated with a high risk of mortality in cancer/COVID-19 patients are older age, ever-smoking status, coexistence of additional comorbidities (e.g., diabetes), and presence of chronic health conditions.

**Figure 1.**
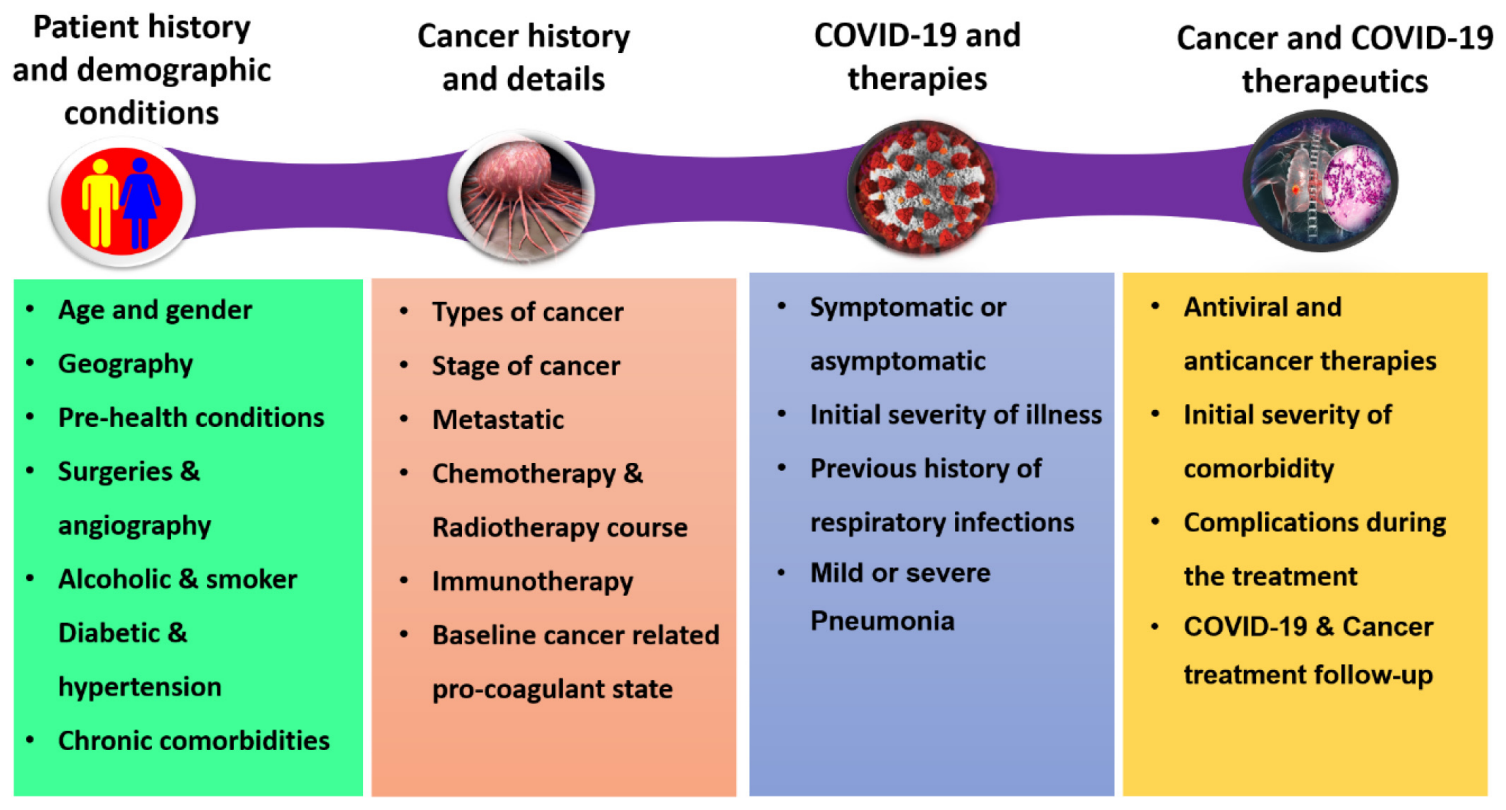
Schematic correlation of cancer and COVID-19 in the current clinical settings.

Cancer patients are more likely to be infected by COVID-19, and clinical outcomes across the globe have evidenced that SARS-CoV-2 infection leads to significant adverse events in oncological patients; as a result, this comorbidity unavoidably reflects an increased mortality rate, and SARS-CoV-2 and cancer have been indicated as real partners of a single crime ^8^. Many national population-based modelling studies support the concept that patients undergoing anticancer therapeutic regimens against all types of solid tumours (including breast, brain, lung, prostate, head and neck, and malignant neoplasms) and haematological malignancies, have a poorer therapeutic response and higher mortality rate after COVID-19 infection ^9^. Potential cohort clinical studies involving cancer patients are currently being evaluated with the purpose of answering the crucial question of whether SARS-CoV-2 virus infections develop extreme or life-threatening complications ^10, 11^. The ability of cancer patients to combat external infections is markedly reduced due to their immunocompromised conditions; therefore, the risk of serious events associated with COVID-19 is remarkably greater in the oncological population. In addition, cancer patients are subjected to a higher risk of SARS-CoV-2 infection not only because of age or other pre-existing life style factors (e.g., smoking) but also because of cancer-associated metabolic disorders and chemotherapy/radiotherapy side effects that might exacerbate COVID-19 symptoms. Because of these pre-existing health conditions, cancer patients require timely diagnosis and faster intensive care admission in comparison to other comorbidities. The schematic correlation of cancer and COVID-19 comorbidity and its consequences in the current clinical settings is shown in Figure 1. 

With the purpose of counteracting the COVID-19 pandemic, strong measures including national lockdowns were introduced by almost all countries in March 2020 and many countries are still imposing major restrictions on citizen mobility. Unfortunately, during lockdown cancer screening campaigns were interrupted, regular diagnostic investigations postponed, and only late stage oncological conditions were scheduled for priority intervention. In addition, hospitals and oncology centres around the world have restricted cancer patient mobility, aiming to limit patient exposure to COVID-19 ^12^. Clearly, setting aside the direct threats to patients with cancer brought about by SARS-CoV-2 infection, late diagnosis and sub-standard oncological therapeutic regimens of care may largely and negatively affect cancer-afflicted individuals ^1^. 

The main focus of this data-driven review is to provide insights to scientists and general readers on the one-year consequences of the COVID-19 pandemic on cancer management. Another purpose is to offer some important criteria that clinical oncologists might follow while making decisions about cancer therapies to be administered under the extra pressure exerted by this unprecedented viral spreading. Furthermore, the impact of the COVID-19 pandemic on cancer management and therapeutic approaches is discussed, as we surmise that the role of multimodal therapies for cancer COVID-19 and new drug developments may have been underestimated so far. Finally, this review covers the concept of anticancer drug repurposing or repositioning in the combined treatment of COVID-19 and cancer comorbidity. The mortality (%) of hospitalized cancer (major) patients due to COVID-19 across the world is given in Table 1.

**Table 1.** Mortality (%) of hospitalized cancer (major) patients due to COVID-19 across the world.

Cancer type	Breast	Brain	Head/neck & oral	Lung	Prostate	Colorectal	Haematological malignancies	Liver	Advanced metastatic	All other
Country										
USA	14.00	13.00	13.00	55.00	15.00	38.00	37.00	38.00	37.00	28.00
India	10.02	5.1	22.20	----	07.40	----	25.90	33.30	37.00	14.52
Brazil	15.70	----	01.90	11.80	07.80	07.80	19.60	15.70	21.00	12.40
UK	15.00	----	02.00	14.00	13.00	-----	22.00	19.00	26.11	43.55
Turkey	05.90	----	----	05.90	23.50	-----	23.00	23.50	69.00	23.94
Italy	22.22	----	----	22.22	33.34	-----	22.22	-----	40.00	36.00
Spain	34.23	----	----	09.51	30.73	30.94	-----		35.00	25.00
France	23.70	----	23.50	41.00	30.30	27.40	33.80	27.00	46.00	27.10
China	18.20	-----	-----	18.20	18.20	-----	41.00	09.10	-----	21.10
Japan	-----	-----	-----	09.09	27.00	09.00	27.00	09.00	-----	34.00
Iran	07.40	-----	-----	-----	07.40	-----	63.00	18.50	-----	50.94
European Union	11.60	-----	-----	17.50	-----	-----	19.12	-----	51.33	11.06
Worldwide	08.28	-----	-----	26.60	05.26	09.00	24.55	-----	-----	25.15

Sources: (i) USA: New York Hospital System, USA ^14^; (ii) India: Rajiv Gandhi Cancer Institute & Research Centre, New Delhi, India ^15^; (iii) Brazil: National Cancer Institute, São Paulo, SP, Brazil ^16^; (iv) UK: a European registry of patients ^17, 18^; (v) Bakırköy dr. Sadi Konuk Training and Research Hospital, University of Health Sciences, Istanbul, Turkey ^19^; Italy: Piacenza’s general hospital (north Italy) ^20^; France: Institut Curie hospitals in the Paris area ^21^ and National hospital chain ^22^, China: Retrospective study based on medical records ^23^; Japan: Tokyo Metropolitan Cancer and Infectious Diseases Center Komagome Hospital, Tokyo, Japan ^24^; Iran: Shariati Hospital, Tehran University of Medical Sciences, Tehran, Iran ^25^, European Union (EU): European registry of patients with cancer consecutively diagnosed with COVID-19 in 27 centres from EU countries ^17^. Worldwide: PubMed, Cochrane Library, and Embase Ovid databases ^26^.

## COVID-19 and cancer: immuno-oncological perspectives

**Figure 2.**
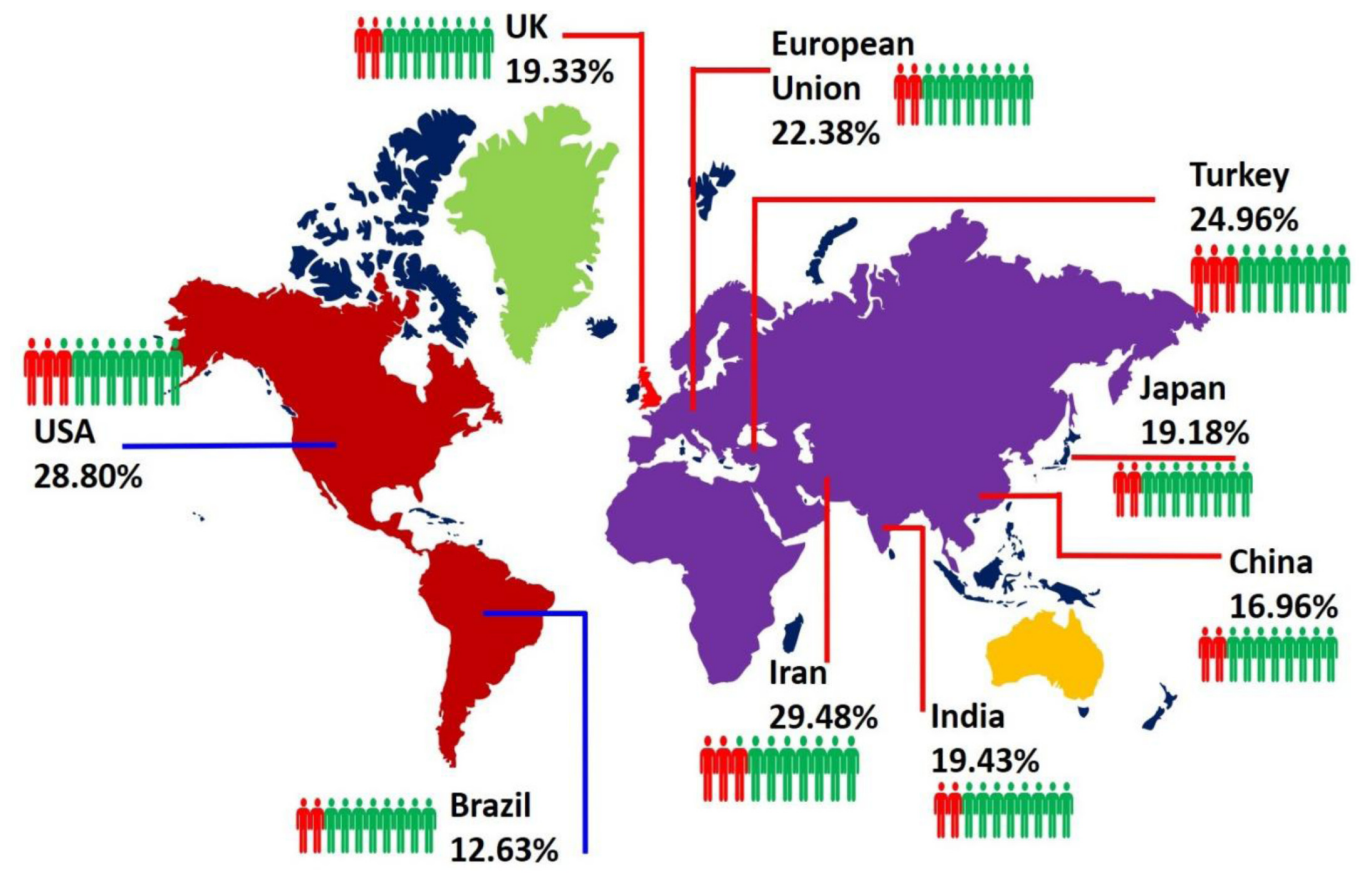
Worldwide scenario of COVID-19 mortality due to major cancers (breast, brain, head & neck-oral, lung, prostate, colorectal, liver and haematological malignancies). The figure is designed from the data collected in Table 1.

Although many of the details of the SARS-CoV-2 virus and its effect on patients who suffer from cancer have still to be clarified, a few remarkable, common features are emerging between COVID-19 pathophysiology and cancerous malignancies. In particular, the onset of inflammation, dysregulation of the immune system and impaired clot formation (also known as coagulopathy) are three hallmarks of both diseases. People living with cancer are more prone to become infected by SARS-CoV-2, and this is strongly linked to their weakened immune system ( Figure 2). Infections with SARS-CoV-2 can elicit host inflammation, leading to cytokine-induced vasodilation, extravasation of neutrophils, and plasma leakage into the affected tissue ^13^. In addition, SARS-CoV-2 replication within infected cells stimulates increased levels of the pro-inflammatory mediators, including several interleukins (e.g., interleukin (IL)-6, IL-1β, IL-2 and IL-10), interferon (IFN)-γ, and tumour necrosis factor (TNF)-α. Within the vast majority of affected organs, the growth pattern of cancer cells is abnormal and uncontrolled, and ultimately these cells leave the primary site tumour to migrate to other body parts, where they originate secondary lesions known as metastases. Current chemo- or-radio-based anticancer therapies rely on the principle of killing fast-dividing cells either by cytotoxic drugs or radiation, with the ultimate aim of controlling metastasis growth and spread. However, other non-malignant rapidly-growing cells – including leukocytes (white blood cells or WBCs) and lymphocytes such as T and B cells ^9^ – can also be killed by such treatments, and this additional attack to the immune system of cancer patients render these individual less responsive to ongoing therapies. Additionally, cancer patients’ weak immunity itself cannot provide the powerful weapon required to face external threats such as viral or bacterial infections. Hence, these patients are undoubtedly exposed to an increased risk of SARS-CoV-2 infection, and have a three to four-fold higher mortality rate due to COVID-19 compared to other non-malignant comorbidities ^27, 28^. 

Several clinical data analysis reports have addressed the incidence of COVID-19 patients with cancer across all continents. Among the cancer types, haematological and thoracic cancers have been associated with worse mortality rates since the beginning of the COVID-19 outbreak ^29^. Recent studies have also shown that type 2-diabetes is linked to deaths from COVID-19 among cancer patients, the majority of affected individuals having liver, pancreatic or endometrial malignant neoplasms ^30^. On the other hand, pre-existing lung comorbidities such as lower respiratory reserve, smoking and age status, and a clinical history of thoracic cancer and or anticancer treatments were shown to be connected with worse COVID-19 outcomes in populations from both USA and China. In detail, reports published from the USA demonstrated that lung cancer patients have a 55% higher risk of mortality from COVID-19 when compared with patients suffering from other cancer types (28%) ^14^, while studies from China reported lung cancer patients are more vulnerable (18.8% mortality) to COVID-19 when compared with other cancer types (11.1% mortality) ^28^.

**Figure 3.**
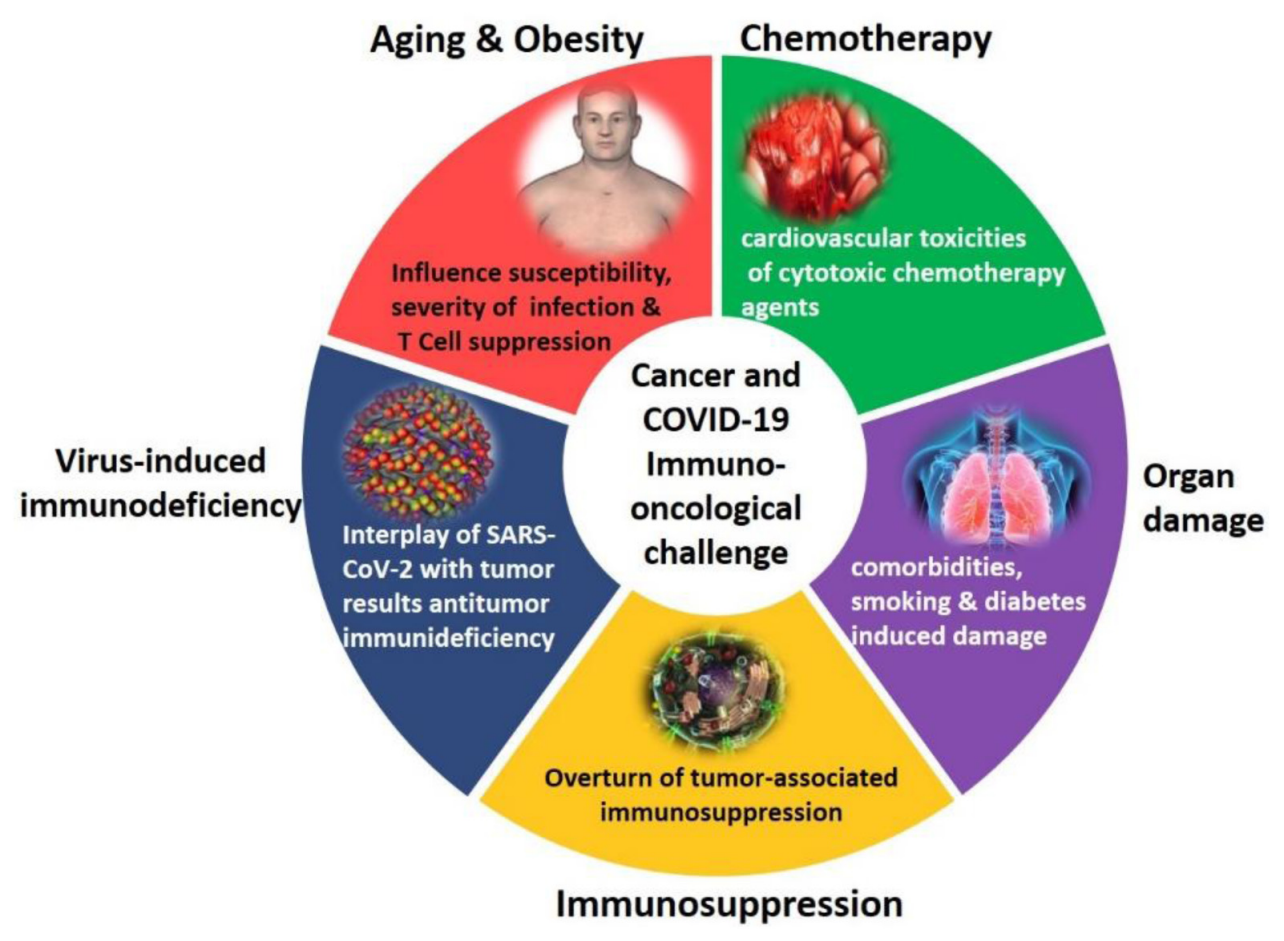
The most prominent therapeutic challenges of cancer patients during the COVID-19 pandemic.

The most prominent therapeutic challenge for cancer patients with COVID-19 is age as shown in Figure 3. Aging is primarily responsible for increasing cumulative mortality incidence within the oncological population, due to the combination of immunosuppressive effects - induced by the disease itself and chemotherapeutic drugs - and physiological immunosenescence. Since SARS-CoV-2 infections already promote systemic changes and damage to the immune system of healthy individuals, its role in weakening the already-waning innate immunity of cancer patients is clearly a prevailing factor compared to non-cancer patients. Thus, the lack of effective immunity to combat SARS-CoV-2 infection is among the prime reasons for high COVID-19-related mortality in oncological patients ^31^. Notably, SARS-CoV-2 infection not only reduces T cell counts but also stimulates polarization of age-induced immune cells and expression of inflammatory-related genes ^30^. Furthermore, aging-associated immune response dysregulation was confirmed in older individuals diagnosed with both COVID-19 and cancer, and this unavoidably concurs both in weakening the host ability to suppress SARS-CoV-2 infections but also to mount effective responses to anticancer therapies, thus leading to the vulnerability of cancer patients to COVID-19 ^32^. Additionally, the generation of naive T cells through thymopoiesis and their priming by tumour specific antigens and SARS-CoV-2 infections are compromised with aging ^33, 34^. Unregulated mitochondrial functions are involved in inflammation and dysfunctional response to SARS-CoV-2 infection ^33^; as a result, older persons are more susceptible to cancer and SARS-CoV-2 infections, as well as being less able to build adaptive immune responses after COVID-19 treatment.

## COVID 19: Compromised cancer management

**Figure 4.**
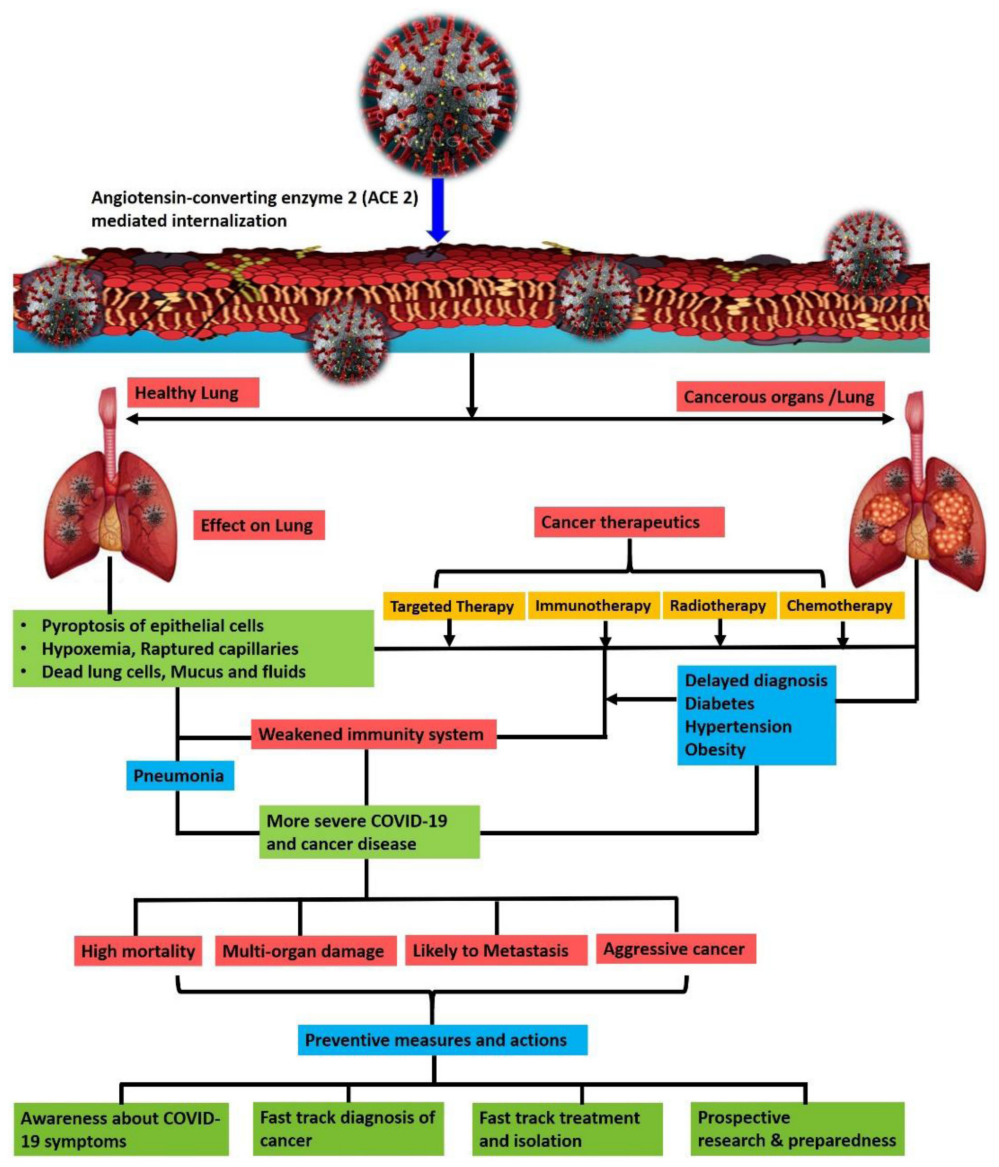
Cancer and COVID-19 comorbidity scenario.

Early on in the COVID-19 pandemic, the World Health Organization (WHO) found that cancer care (including diagnosis and therapy) has been severely negatively impacted in more than 150 countries worldwide ^35^. Figure 4 shows the cancer and COVID-19 comorbidity scenario. Contextually, all European nations and the United States reported partial or complete interruption of cancer services. For instance, a 46.4% decrement in cancer diagnosis protocols was reported in the first month of COVID-19 outbreak in the USA (March 2020) ^36^. In the European territory, many national health services also reported a substantial decline in the number of cancers diagnosed at the beginning of the COVID-19 pandemic (e.g., the Netherlands and Belgium reported a 30–40% decline in diagnosis, while the UK reported a decline of up to 84%) ^37^. Similarly, India reported a 50% decline ^38^ in new diagnoses; Japan, Korea, Canada and Australia reported declines of nearly 45%, and China along with African countries reported a 10% decline in March–April 2020 in new diagnoses of cancer ^39, 40^. Such delayed cancer diagnosis and treatments are projected to result in an increase in the number of cancer-related deaths, in particular from colorectal cancer (+15%) and breast cancer (+9%) over the next five years ^11^. Under this perspective, the concrete fear of a further medical emergency linked to noncommunicable diseases (including cancer) brought on by the pandemic is currently emerging.

The impact of COVID-19 on cancer throughout the world is complex, and has been referred to as a “deadly interplay” by WHO ^35^. Unexpected travel restrictions, as well as the risk of SARS-CoV-2 infection among patients, have interrupted cancer services around the world, reducing the odds of a cure or survival for millions of cancer patients. Another issue in cancer management is drug shortages, which have resulted in a considerable decline in the number of new diagnoses and pre-planned therapy, even in nations with the strongest health-care systems. Treatment is extremely expensive for both COVID-19 and cancer, posing a challenge to the health-care systems of all emerging, developing, and high-income countries.

Throughout 2020, data from multiple cohort studies revealed that lung cancer was the most common malignancy linked to poor outcomes during the COVID-19 pandemic ^41, 42^. Among these patients, persons over the age of 60 are more likely to have lung cancer and SARS-CoV-2 comorbidity in comparison to their younger counterparts. One of the prevalent reasons for this outcome is the SARS-CoV-2 host-cell entry mechanism. Indeed, this pathogen targets lung tissue and enters lung cells via the angiotensin-converting enzyme 2 (ACE2) receptor. The viral load overwhelms other organs over time, leading in a cytokine storm and organ crosstalk that is dependent on the expression of the ACE2 receptor. Lung cancer patients have symptoms comparable to COVID-19 patients, such as systemic inflammation, fever, cough, and shortness of breath, as well as additional cancer consequences that can lead to multi-organ failure; thus COVID-19 mortality is more likely in this comorbidity ^43^. Following lung cancer, other blood, bone marrow, and lymph node (haematological) malignancies were linked to increased vulnerability and accounted for up to 10-39% of global mortality from SARS-CoV-2 infections ^44– 46^. The characteristics of lung and haematological cancer patients, for example haemoptysis, loss of weight, loss of appetite, dyspnoea, thoracic pain, their age and the co-existence of further chronic diseases (both known or unknown), set further hurdles in any of these malignant clinical settings, with the COVID-19 pandemic resulting in much worse outcomes ^47^. In line with this, patients with a haematological malignancy and lung cancer have an elevated risk of a poor outcome of COVID-19 in Europe and other parts of the world, according to studies carried out throughout the year 2020 by the Dutch Oncology COVID-19 group in the Netherlands ^17, 48– 52^. As a result, the advice to clinicians with these vulnerable individuals as patients is to minimize their exposure to SARS-CoV-2 during treatment changes and, when possible, immunization should also be considered for continuing cancer patients.

**Figure 5.**
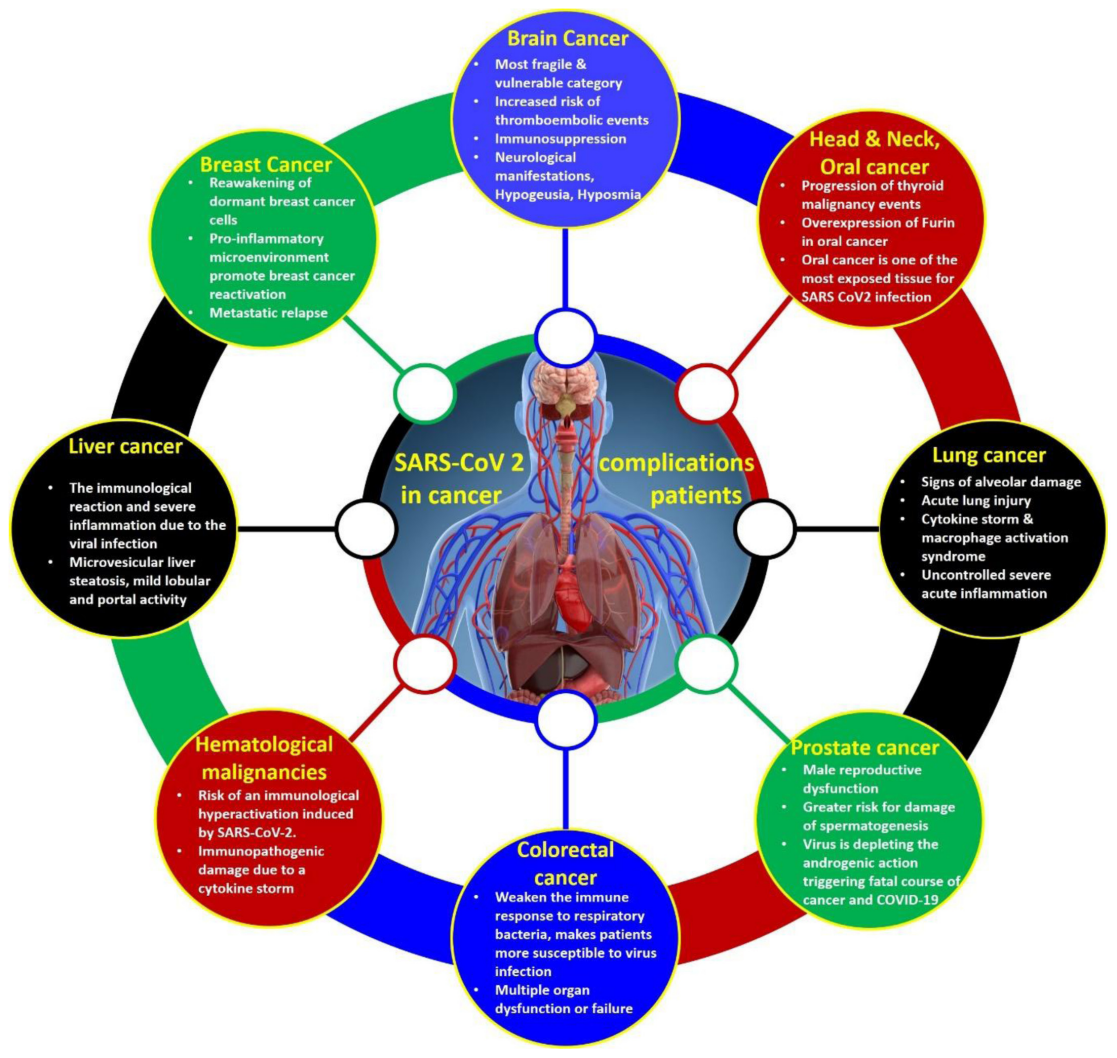
Additional side effects of SARS-CoV-2 infections in cancer patients, the cancers that represented the highest mortality from COVID-19 since March 2020 and cancers that represent aggravating comorbidities of COVID-19. SASS-CoV-2 infections trigger additional oncological challenges and therapeutic burdens to cancer patients.

Increased mortality among older adults is also associated with infection anxiety, leading to delayed hospital admissions and other supportive care ( Figure 5). In addition, after the COVID-19 outbreak, more than 34% of cancer surgical procedures were cancelled, and reduced monitoring and normal follow-up for adverse reactions to chemotherapy has suppressed therapeutic plans for older persons around the world ^53^. According to the European Commission report on cancer management and COVID-19 ^54^ in the European region, early cancer diagnosis awareness campaigns were either suspended or cancelled, not only in older patients but also in younger patients. Breast and cervical cancer-screening services in women and vaccination programmes (e.g. against human papillomavirus, HPV) continued during the first wave of COVID-19. This has been proposed in response to an impending health crisis caused by an overabundance of cancer patients, which is predicted to increase the strain on health systems globally, not just in Europe.

## Associations of COVID-19 and cancer therapies

The management of cancer patients during the COVID-19 pandemic is mainly based on the provision of adjunctive support, including antiviral drugs, oxygen therapy, chemotherapy and radiotherapy, which represents the main treatment course. Despite the fact that novel anti-SARS-CoV-2 curative medications have yet to be discovered, the administration of anti-inflammatory, anticancer, and antiviral therapy is currently recommended ^55^. WHO has recently updated interim guidance for cancer comorbidity, with the major aims of designing standard care to: (i) attenuate and prevent COVID-19 transmission in cancer patients; (ii) develop new antiviral and anticancer therapy protocols that can provide optimal care for all patients; (iii) develop fast-track solutions to promote effective multiple treatments; and (iv) ensure cancer research and care continuity while battling COVID-19 ^35^.

An association between cancer and SARS-CoV-2 infections, as well as a direct relationship between SARS-CoV-2 and cancer therapeutics, is still not well studied and elaborated in the literature. As discussed above, the weakened immune system of cancer patients is a key player in the individual outcome of SARS‑CoV‑2 infection. Many immune boosters, antiviral medications, and reciprocal anticancer medications with possible multifunctional therapeutic qualities have recently been examined for mild to severe COVID-19 cases, based on a number of clinical cohort studies and data from cancer patients with COVID-19 ^56^. A higher viral load, which may favour immunosuppression, also predisposes cancer patients to more intense viral replication ^57^. The activation of T cells, which detect and destroy SARS-CoV-2-infected cells, is required for a powerful immune response against COVID-19. Additionally, T cell activation by cancer therapy also improves the ability of our own immune cells to fight cancer. This dual nature of T cells drew the attention of researchers and physicians, who devised a treatment protocol applying T cell-activated immunotherapy to this comorbidity. In contrast, recent observational clinical studies in oncological cohorts with confirmed severe COVID-19 comorbidity reported a reduced number of CD4 ^+^ cells (i.e., T-helper cells active against infections) and CD8 ^+^ cells (specialized in killing cancer cells and other antigens) ^58^. Because T cells play such an important role in virus-infected cells, having a larger functionally protected T cell pool may help to lessen the severity of COVID-19 in cancer patients. This led to the development of integrated immunotherapy protocols for cancer and COVID-19 patients, which included immune-checkpoint blockers (pembrolizumab, nivolumab, cemiplimab) that might increase T cell immune responses against SARS-CoV-2 infected cells and malignant cells at the same time ^59^. Encouragingly, recent retrospective cohort studies in haematological cancer patients with COVID-19 demonstrated positive outcomes of immunotherapy and endocrine therapy ^60^. These clinical data strongly suggest that immunotherapy is somewhat beneficial to cancer patients with COVID-19. Similarly, large-cohort studies from the UK Coronavirus Cancer Monitoring Project and CCC19 reported that immunotherapy is advantageous to control COVID-19 in cancer patients ^61^. In contrast, in a study of 275 patients, undergoing immunotherapy, particularly among lung cancer patients, was linked to an increased likelihood of intensive care unit (ICU) admission ^62^. As a result, extended immunotherapy could be a big issue for COVID-19 cancer patients, as immune-mediated pneumonitis is a common adverse effect. In addition, clinical protocols involving steroid-based therapies for treating cancer/SARS‑CoV‑2 infection comorbidities have become established trends in the management of COVID-19 ^63– 65^.

As of today, COVID-19 is being treated with a class of anticancer medications with antiviral characteristics that may raise or decrease vulnerability to SARS-CoV-2 infections. Administration of anticancer treatments to COVID-19 patients carries the risk of toxicity and infection severity due to the immunosuppressive nature of anticancer medications. As a result, numerous chemotherapeutic medications have been linked to an increased chance of severe COVID-19 ^56^, while infected individuals who received immune checkpoint inhibitors or targeted anticancer chemotherapeutics were shown to be more resistant to SARS-CoV-2 infections, whether mild or severe ^56^. In rare cases, oncological patients subjected to extensive chemotherapy with various medications may experience immune-related side effects of the lung including pneumonia; clearly, this constitutes an additional risk factor in cancer patients impacted by COVID-19 and chemotherapeutic regimens.

New cancer chemotherapeutics are improving the host immune system's ability to recognize and eliminate cancer cells. The strong link between oncology drugs that enhance antitumor immune responses and promote better outcomes in SARS-CoV-2 infected individuals is now convincingly demonstrated by several clinical cohort studies, as shown in Table 2. Intrinsic immune responses induced by anticancer therapies confer extra antiviral immunity and are critical for developing optimal COVID-19/cancer comorbidity therapeutic strategies. Antiviral immunity is also efficiently triggered by the delivery of chemotherapeutics in patients with a cancer burden via *in situ* techniques ^66^. Chemotherapeutic medicines have long been known for their anticancer properties, but they are now also being recognized for their antiviral and immunotherapeutic properties. During SARS-CoV-2 infection, the effects of antiviral immune responses can be amplified using chemotherapeutics via the subsequent immune cascade; indeed, the internalization of chemotherapeutics into the bloodstream and lymphatic system activates the first line immune response as well as recruits second line responders (e.g., neutrophils and macrophages) to the infection site. Hence, the efficacy of antiviral therapy is dependent on the recruitment of immune cells caused by the anticancer chemotherapeutics. Beside anticancer drugs, other antiviral, antibiotic, and antimalarial active principles - along with plasma therapy - have also been tested to avoid severe SARS-CoV-2 infections ^56^. Regardless of a lack of strong clinical evidence, multiple COVID-19 treatment protocols have been adopted in clinical practice across the world. Interestingly, some preliminary findings show promising results for these protocols against COVID-19 and cancer in comorbid patients. Although clinical trials remain the mainstay practice in cancer treatment, in the current COVID-19 pandemic drug repurposing can be a compelling and promising method to control SARS-CoV-2 infections.

**Table 2.** Drug design and potential therapeutic approach of repurposing for COVID-19.

Dual therapeutic Drugs	Mode of action on COVID-19	Mode of action on cancer	Type of study ^(ref)^
Anakinra	Selectively targeting inflammatory cytokine (IL-1), shown to be a safe and effective approach to avoid mechanic ventilation in hospitalized patients with moderate to severe COVID-19 pneumonia.	Blocking an inflammatory cytokine (IL-1β) that diverts self-aggravating inflammation and reduces glioblastoma aggressiveness.	Patients with severe COVID-19 have less need for invasive mechanical breathing and have a lower mortality rate, with no major side effects ^69^.
Azithromycin	Azithromycin may raise the pH of the Golgi network and recycling endosome, interfering with intracellular SARS-CoV-2 activity and reproduction, and reducing the virus's capacity to infect cells.	Inhibits cancer cell growth while also selectively targeting tumour cells and initiating tumour necrosis factor-related apoptosis-inducing ligand (TRAIL) activities.	Numerous randomised clinical trials with over 100 individuals provided data on the efficacy of azithromycin as a COVID-19 therapy ^70^.
Baricitinib	Inhibits the intracellular signalling pathway of cytokines known to be elevated in severe COVID-19	Selective inhibitor of Janus kinase (JAK) 1 and 2	Double-blind clinical trial against COVID-19 shows positive results ^71^.
Bevacizumab	Vascular endothelial growth factor (VEGF) inhibition. Blocking VEGF and its associated signalling would increase oxygen perfusion and anti-inflammatory response, as well as ameliorate clinical symptoms in individuals with severe COVID-19.	Selectively binds to circulating VEGF, blocking VEGF's binding to its cell surface receptors, resulting in a reduction in tumour blood vessel microvascular development and thereby limiting blood flow to tumour tissues.	During a 28-day follow-up, a single-dose therapy improved oxygen-support status in 92% of patients, with no deaths ^72^.
Carfilzomib	Blocking of viral replication.	Proteasome inhibitor that selectively and irreversibly binds to its target and down- regulates proto-oncogenic nuclear factor kappa B (NF-кB), promoting tumour cell apoptosis.	Recommended for management of COVID- 19 patients with multiple myeloma during the COVID-19 pandemic ^73^.
Chlorpromazine	The pharmacokinetics of chlorpromazine increase its concentration in the lungs (20-200 times greater than in plasma), which is beneficial in reducing SARS-CoV-2 contagiousness. It can also cross the blood-brain barrier, thereby preventing COVID-19 neurological manifestations.	Chlorpromazine enhances the effect of other anticancer drugs such as tamoxifen through an oestrogen receptor-mediated mechanism.	Large clinical trials on COVID-19 patients have shown that it is effective in treating patients with severe COVID-19 ^74^.
Duvelisib	Immune homeostasis restoration and inhibits phosphoinositide-3 kinase δ and γ, thus controlling viral replication.	Duvelisib is a dual inhibitor of phosphoinositide- 3-kinase (PI3K)-δ and PI3K-γ. In hematologic malignancies, PI3K- δ is constitutively expressed, and its inhibition has been found to limit the proliferation of several hematologic tumour cells while allowing normal immune cells to survive.	Numerous randomised clinical trials with COVID- 19 patients produced data on the efficacy of duvelisib as a COVID-19 supportive medication ^13^.
Hydroxychloroquine (HCQ)	SARS-CoV-2 endocytic pathway, sialic acid receptor blocking, pH-mediated spike (S) protein cleavage at the angiotensin-converting enzyme 2 (ACE2) binding site, and cytokine storm prevention are all affected by HCQ.	Autophagy sustains the survival of malignant cells. HCQ has been shown to mediate substantial inhibition of autophagy.	The results of a comprehensive clinical trial are inconclusive in favour of its usage in COVID-19. Given the observational design and the 95%confidence interval, there was no evidence of either benefit or harm from HCQ treatment ^75^.
Imatinib mesylate	Inhibits kinase activity involved in coronavirus fusion with endosomal membrane and late infection cell–cell fusion. SARS-CoV-2 replication is thus slowed, owing to the suppression of the Abl2 protein kinase activity.	Imatinib targets selective tyrosine kinase inhibitors (TKIs) and deregulates protein tyrosine kinase activity that is central to the pathogenesis of various human cancers.	Recommended for the treatment of SARS-CoV- 2 infection and randomized controlled study in COVID-19 patients with moderate and severe pneumonia are ongoing ^76^.
Pembrolizumab, nivolumab	Mitigate lymphocyte exhaustion and defective activation of T lymphocytes caused by SARS-CoV-2 infection.	Selectively binds to the programmed cell death-1 (PD-1) receptor on T cells, deactivating a negative regulatory signal of T cells, thereby furthering their antitumor activity.	Pembrolizumab was given to COVID-19 patients in phase 3 trials at a dose of 2 mg/kg every three weeks and resulted in decreased SARS-CoV-2 infections ^77^.
Toremifene	SARS-CoV-2 viral replication is controlled by toremifene, a selective oestrogen receptor modulator that suppresses the spike glycoprotein, which is responsible for assisting in the fusing of the viral membrane with the cell membrane via a disruption to the fusion core.	Selective ability to compete with oestrogen for binding sites in tumours, preventing oestrogen's growth-stimulating effects. It also inhibits tumour growth by inducing apoptosis, controlling oncogene expression, and blocking growth factors.	COVID-9 FDA-approved drug that blocks the Spike glycoprotein and SARS-CoV-2 NSP14 ^78^.
Virafin Interferons (IFN-beta)	Interferons are growth inhibitory compounds that can operate as antivirals in the host defence system by destroying virally infected cells and triggering the transcription of interferon-stimulated genes, which perform numerous antiviral roles.	Virafin works as an immune stimulant, boosting T helper type 1 (Th1) cell responses, increasing MHC class I expression, and inducing death in cancer cells via natural killer (NK) cell and T-cell mediated cytotoxicity.	Data from clinical investigations (including a cohort of 50 COVID-19 patients) revealed that multiple interferon molecules may be a defining feature of severe COVID-19 and provide support for combination therapy approaches ^79^.
Zanubrutinib	Immune, fatal, and sepsis-induced pulmonary damage are all protected against. Zanubrutinib works by inhibiting Bruton’s tyrosine kinase (BTK), which is involved in the generation of a variety of proinflammatory cytokines. Reduced cytokine levels arise from inhibiting BTK signalling, which has an anti-inflammatory effect.	Zanubrutinib is a targeted anticancer drug that acts as an immunomodulatory chemotherapeutic cargo by inhibiting particular molecular abnormalities that enable oncogenesis or tumour development.	Clinical studies (cohort) on COVID-19 patients suggest that BTKi (zanubrutinib) may have protective action against SARS-CoV-2 ^80^.
Chimeric antigen receptor (CAR) T cell therapy	CAR T cells can be redirected to target the SARS- CoV-2 by either a CAR or a T-cell receptor (TCR) that can recognize SARS-CoV-2 pathogens, and then guiding the immune cells to eradicate the targets when infused back into the patients.	T cells that have been genetically modified to express a CAR are infused using this therapeutic method to reprogram T cells. The CAR binds cancer cell surface antigens and kills tumour cells using a single-chain variable fragment, which combines the specificity of a monoclonal antibody with the cytotoxic and memory abilities of T cells.	Recommended for the treatment of COVID- 19 patients with cancer during the COVID-19 pandemic ^81^.
Low-dose radiotherapy	External beam irradiation, single fraction of 0.5 Gy can act as immunomodulation reprogramming, and controls lung inflammation induced in SARS-CoV-2 viral pneumonia.	By damaging cancer cells' DNA, radiation therapy kills or slows cancer cells' growth. Cancer cells that have had their DNA damaged beyond repair will either cease proliferating or die. When damaged cells die, they are broken down and disposed of from the body.	Low-dose radiation (0.5 Gy) is an evidence- based anti-inflammatory treatment that has the potential to change the immunological landscape in COVID-19 patients' damaged lungs ^82^.

## Assessing the road ahead

To create an evidence-based therapeutic approach and to build clinical protocols for treating patients affected both by cancer and SARS-CoV-2 infection, a systematic clinical investigation of the implications of acute COVID-19 and malignant neoplasms is required. In addition, a comprehensive understanding of comorbid patient care required beyond the acute phase of COVID-19 is urgently needed. New therapy protocols combining anticancer, antiviral, antifungal, and other supporting medications will aid in providing integrated multispecialty patient care. The most recent clinical cohort studies discussed in this review suggest that managing cancer patients infected with SARS-CoV-2 requires a substantial revision of current pharmacological treatments. Furthermore, the interpretation of cancer-relevant mechanisms engaged by the SARS-CoV-2 virus is essential for evidence-based cancer therapeutics, while the links between SARS-CoV-2 infection, tumorigenesis, and cancer patient immunosuppression must be predicted in epidemiological settings. Contextually, the adoption of a highly advanced approach such as those based on the application of artificial intelligence (AI) to COVID-19/cancer comorbidities could be highly beneficial, as AI can reduce the timeline of new protocol development, a critical issue in the current health emergency ^67^. During such an evolving and dramatic period, AI could not only decrease development time but also the costs of these therapeutic strategies, allowing for equal treatment options to be available worldwide. AI could use cutting-edge computational algorithms and clinical data for network medicine, which allows existing drugs to be tested across multiple diseases. In the big-data age, AI-guided drug repurposing or repositioning can overcome illness severity using early prognostic platforms, making it possible to define disease, medicine, therapies, and target identification with the least amount of mistakes in a very short amount of time.

**Figure 6.**
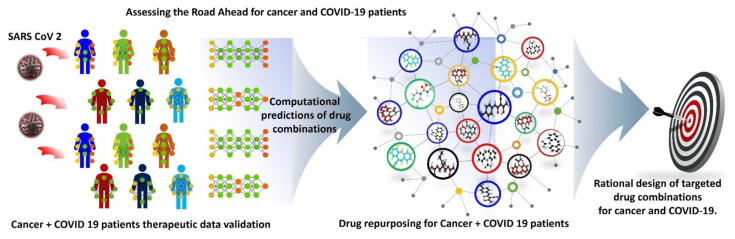
Assessing the road ahead for cancer and COVID-19 patients using an artificial-intelligence-based rational design of drug combinations.

At the current time, a network-based method for the systematic identification of targeted therapeutic and drug combinations for a prospective treatment of COVID-19 and cancer is a matter of urgency, given the lack of complete illness information about COVID-19 and its effects on cancer patients. Integration of drug and SARS-CoV-2 target networks, virus-host interactions, cancer induced immunosuppression in patients and antiviral-anticancer drug interactome networks are essential for such identification ( Figure 6). The current pharmacology model of anticancer drugs used to treat COVID-19 cannot separate antiviral-anticancer effects from those predictions due to a lack of detailed pharmacokinetics of drug targets and consequences of virus–host interactions ^68^. The wide- ranging biological effects of different drug combinations as well as drug repurposing for both diseases will significantly improve the accuracy of the treatment combinations for the quick recovery of COVID-19 patients.

Early reports of COVID-19 outcomes in cancer patients have been largely derived in retrospect from small cohorts of SARS-CoV-2 infected individuals. Therapeutic conclusions, such as an increased risk of death from immunotherapy and chemotherapy, were taken from a small number of clinical studies and patient subgroups (as an understandable obvious consequence of COVID-19 constraints). Much of the research discussed in this article shows that systemic chemotherapy treatment choices for higher-risk SARS-CoV-2 infected patients are limited, which may lead to poorer results rather than curative treatment. While anticancer drugs and chimeric antigen receptor (CAR) T cell therapy summarized in Table 2 could indeed be effective in combatting the cytokine storm induced by COVID-19, the risk associated with some of these treatments is controversial due to reported side effects of fever, malaise and myalgias, severe organ toxicity, lung failure ^83^. Anti-cancer medications and other immune-modulating active principles, whether used as a supplement or as part of a cancer therapy plan, may have different effects in COVID-19 patients; moreover, SARS-CoV-2 infections are being treated with other medicines that are routinely used in cancer therapeutic settings (e.g., steroids) that, in turn, affect host immunity ^84^. An excessive usage of steroids in COVID-19 patients, however, was recently linked to a mucormycosis outbreak in India: according to media sources, hundreds of COVID-19 patients perished in India alone in the third week of May 2021 owing to mucormycosis fungal infection following steroid-based anti-SARS-CoV-2 treatments. This clearly highlights the necessity of low-dose steroidal regimens in managing cancer/COVID-19 patients during the present and possible future health emergencies. In order to reduce risk and enhance cancer treatment results, clinicians and policymakers must possess an essential, multidisciplinary knowledge of the complex and delicate interplay of SARS-CoV-2 virology, tumour biology, immune system physiology and anticancer therapeutic mechanisms in order to operate in the wider context of global healthcare.

## Conclusion: key recommendations for safeguarding SARS-CoV-2 and cancer management

One of the most important questions that physicians and researchers have had to answer was whether COVID-19 patients with cancer comorbidity have worse outcomes owing to chemotherapeutic drugs than patients who were not treated with the same medications. The definitive answer to this question will aid clinicians in developing novel COVID-19 treatment protocols based on life-saving chemotherapeutics or antiviral drugs. Many national and international health authorities including the European Centre for Disease Prevention and Control (ECDC) ^85^, the European Medicines Agency (EMA) ^86^, the United States Center for Disease Control and Prevention (CDC) ^87^, the National Health Service (NHS) in the UK ^88^, and the National Institute of Infectious Diseases (NIID) ^89^ in Japan issued protocol guidelines for the treatment of patients affected by cancer/COVID-19 comorbidity ( Table 3). All authorities specifically advise that chemotherapeutic, antiviral, and steroid doses be used in a controlled manner. Furthermore, continued usage of investigational drugs must be context-dependent and adhere to the highest clinical trial standards.

**Table 3.** Key safeguarding recommendations by international health authorities for cancer and COVID-19 patients for management of both diseases.

● Avoid shortages in COVID-19 supportive medicines, anticancer drugs, antiviral drugs, equipment such as ventilators, medical oxygen, and medical staff by investing in national-international production units and research programmes. ● Invest in building more resilient and equal health systems for infectious disease and crisis preparedness across the world. ● Increase multinational research consortia for cancer prevention programmes and improve other cancer services. ● Handle the mental health challenges related to lockdowns and imposed restrictions and include it as an integral part of COVID-19 and cancer care. ● Promote the use of new generation technological solutions in COVID-19 and cancer medicines. ● Devise new 'holistic' approaches to fight cancer and COVID-19 together using a single treatment modality. ● Invest in telemedicine and digital pathology for early diagnosis of cancer, testing support for COVID-19, evidence-based screening programmes for cancer and COVID-19 together with early diagnosis initiatives. ● Provide better technical support for health care professionals for daily clinical practice. ● Increase the currently limited data to examine the effect of COVID-19 in outpatient cancer settings, hence support researchers to publish clinical evidence. ● Prioritise cancer surgery according to the urgency of surgical care patient benefit. ● Establish data registry and monitoring of comorbid cases, cancer stage, severity of SARS-CoV-2 infection and treatment in real-time to benchmark performance and respond to system stresses. ● Adopt evidence-based therapeutic pathways such as combinations of multiple drugs to improve recovery of patients with cancer and COVID-19. ● Establish data monitoring pilot projects caring for patients with cancer during COVID-19 that will help to establish curative models and predictors for high-risk populations during future epidemiological outbreaks or pandemics.

The clinical world is speeding up the therapeutic inventions of many treatments to combat COVID-19, and all authorities have warned about the dangers of such haste, suggesting appropriate safety monitoring and in-depth preclinical study of the investigational product before conducting clinical trials on patients. Converting physical visits for therapy consultation to phone or video calls, and maintaining the highest standards of clinical safety during cancer surgeries, radiation, and chemotherapy treatments are all practical methods that could reduce viral spreading within the oncological population.

## Data availability

No data are associated with this article.
