# Supplementary material for: Safeguarding COVID-19 and cancer management: drug design and therapeutic approach
Source: Open Res Eur. 2021 Jul 5;1:77. [Version 1] doi: 10.12688/openreseurope.13841.1 (PMC10445946; doi:10.12688/openreseurope.13841.1)
Supplement: Peer review history

# Peer review: Reviewer response for version 1

**DOI:** 10.21956/openreseurope.14922.r27220

**Published:** 2021-08-11

**Authors:** Nitesh Kumar

**Affiliations:** 1 Department of Pharmacology and Toxicology, National Institute of Pharmaceutical Education and Research, Hajipur, Bihar, India

The article is nicely written and very relevant to the current scenario. COVID-19 is disrupting immunity and immunotherapy is the fourth pillar for cancer management.  Even chemotherapy in cancer management works as an immunosuppressant. Thus, it is very relevant to study the impact of COVID-19 in the treatment of cancer. The article has discussed all the relevant points related to COVID-19 and cancer management. I must congratulate the authors for highlighting all the relevant points.

 I have a minor suggestion:

- A discussion is required in terms of mortality before COVID-19 and after the COVID-19 outbreak. It will be useful to know that COVID-19 has increased the death rate or decreased the death rate in hospitalized patients.

Is the review written in accessible language?

Yes

Are all factual statements correct and adequately supported by citations?

Yes

Are the conclusions drawn appropriate in the context of the current research literature?

Yes

Is the topic of the review discussed comprehensively in the context of the current literature?

Yes

Reviewer Expertise:

Anticancer Research

I confirm that I have read this submission and believe that I have an appropriate level of expertise to confirm that it is of an acceptable scientific standard.

# Peer review: Reviewer response for version 1

**DOI:** 10.21956/openreseurope.14922.r27219

**Published:** 2021-08-02

**Authors:** Takuro Niidome

**Affiliations:** 1 Faculty of Advanced Science and Technology, Kumamoto University, Kumamoto, Japan

This review manuscript summarized current situation of cancer patients infected with SARS-CoV-2, their managements with controlling immune system, and future prospects. It will be a lighthouse in the chaos world situation for not only clinicians treating the comorbidity but also researchers who develop effective drugs and treatment strategies without adverse effects. However, a point indicated below should be considered before indexing.

 The figures/tables and the explanations in the main text do not match. The figures/tables must be explained politely in the main text. For example, Figure 2 shows 'Worldwide scenario of COVID-19 mortality due to major cancers'. However, in the main text, the authors mentioned that 'People living with cancer are more prone to become infected by SARS-CoV-2, and this is strongly linked to their weakened immune system (Figure 2)'. How can we relate the mortality and the weakened immune system?

Is the review written in accessible language?

Yes

Are all factual statements correct and adequately supported by citations?

Yes

Are the conclusions drawn appropriate in the context of the current research literature?

Yes

Is the topic of the review discussed comprehensively in the context of the current literature?

Yes

Reviewer Expertise:

medical materials, drug delivery system

I confirm that I have read this submission and believe that I have an appropriate level of expertise to confirm that it is of an acceptable scientific standard.
